# Evaluating meaningful impact of Patient and Public Involvement: A Q methodology study among researchers and young people with a chronic condition

**DOI:** 10.1111/hex.13418

**Published:** 2021-12-28

**Authors:** Femke van Schelven, Hennie Boeije, Jany Rademakers

**Affiliations:** ^1^ Netherlands Institute for Health Services Research Utrecht The Netherlands; ^2^ Department of Family Medicine, Care and Public Health Research Institute (CAPHRI) Maastricht University Maastricht The Netherlands

**Keywords:** adolescence, chronic disease, impact, involvement, meaningful, patient participation, Q methodology

## Abstract

**Introduction:**

Although Patient and Public Involvement (PPI) of young people with a chronic condition (YPCC) is receiving increasing attention, evidence of impact is lacking. This is partly due to inadequate understanding of what meaningful impact entails. This study aimed to gain an in‐depth understanding of researchers’ and YPCC's perspectives on meaningful impact.

**Methods:**

We conducted a Q methodology study in a group of 26 researchers and a group of 20 YPCC with experience in PPI. Participants ranked statements about impact (e.g., ‘YPCC acquire new knowledge and skills’) based on their agreement with them. During interviews, they reflected on their rankings (Q sorts). Factor analysis was conducted to identify similar patterns in the individual Q sorts. The interviews were used to determine and interpret the final factor solution. The resulting factors represented distinct perspectives on meaningful impact.

**Results:**

Four distinct perspectives on meaningful impact of PPI were identified. Two were predominantly based on the Q sorts of researchers, for example improving research quality and facilitating dialogue and understanding, and two on the Q sorts of YPCC, for example achieving equality and inclusivity and doing justice to YPCC's rights. The factors were defined by 37 Q sorts (80%); 9 Q sorts did not load significantly on any of the factors.

**Conclusion:**

The results indicate that researchers and YPCC can have different views about the meaningful impact of PPI. The perspectives identified here can serve as an aid when discussing these different views and formulating operational indicators of impact.

**Patient or Public Contribution:**

An adolescent with a chronic condition was involved in the early phases of this study. She helped in formulating the statements and recruiting YPCC.

## 1 INTRODUCTION

Young people with a chronic condition (YPCC) generally play a passive role in research. They are invited to participate in questionnaires and interviews. There is, however, growing consensus that YPCC should be actively involved in research that concerns them. This is also termed ‘Patient and Public Involvement’, or PPI. Hart defined PPI of young people as ‘the process of sharing decisions which affect one's life and the life of the community in which one lives’.^1^ INVOLVE—the former PPI advisory group of the British National Institute for Health Research, now replaced by the Centre for Engagement and Dissemination—devised a general and more practical definition: PPI is about ‘research being carried out “with” or “by” members of the public, rather than “to”, “about” or “for” them’.^2^

Many researchers and YPCC are currently struggling with how to do PPI in research.^3^ PPI can take many forms, with YPCC being involved in various ways and in several stages of various types of research.^4^, ^5^ The literature on PPI of YPCC provides a plethora of examples, from intensive partnerships with a few YPCC^6^, ^7^, ^8^ to consultations with advisory panels.^9^, ^10^ The flexibility of PPI makes it possible to adapt it to the research and the people involved.^11^ At the same time, it complicates PPI practice, since there is no ‘blueprint’ for doing it right.^5^, ^12^ PPI with YPCC is further complicated by the tendency to view YPCC as vulnerable and inexperienced and to underestimate their competence as decision‐makers.^1^, ^3^, ^6^, ^12^ This exacerbates the general issue of power imbalances in PPI.^3^

In recent years, there has been a rising demand for demonstrating the impact of PPI. The literature on PPI of YPCC shows that impact can take many forms.^4^, ^5^, ^11^, ^13^ It is suggested that PPI can increase the relevance and quality of research. YPCC are able to provide new insights from their lived experience, which can improve aspects such as research design,^14^, ^15^ data collection^6^, ^14^ and dissemination of results.^14^, ^16^ PPI is also thought to have a positive impact on the personal development of the YPCC involved, since they learn new skills and gain more self‐confidence.^14^, ^17^ Among researchers, PPI can increase their commitment to research^16^ and evoke feelings of inspiration and pride.^14^ Finally, some research suggests that PPI can enhance the position of all YPCC in society by promoting their right to be heard and supporting inclusivity.^14^, ^18^

Demonstrating the impact of PPI of YPCC can help resolve some of the complexities of PPI.^5^ It provides insight into the achievements of PPI. In addition, demonstrating impact in relation to specific PPI approaches can help identify the PPI approaches that are most likely to realize these achievements. In the words of Staniszewska and colleagues: ‘it requires evidence to inform best practice’.^19^ However, best practice can solely be informed by evidence that is robust and of high quality. Researchers in the field of PPI are currently struggling to find ways to conduct robust evaluations of PPI of YPCC. Several literature reviews—published during a time span of 16 years—have shown that the current evidence base on PPI of YPCC and its impact is weak.^4^, ^11^, ^20^ In recent decades, limited progress has been made to change this.^4^

This limited progress is partly the result of poor understanding of what meaningful impact exactly entails.^21^ A previous study reported that researchers and YPCC find it difficult to specify the impact they achieved beyond general descriptions.^22^ A more detailed understanding of impact can extend our knowledge about the difference that PPI can make and which approaches are effective. We therefore conducted a systematic and extensive study of the perspectives of researchers and YPCC on meaningful impact. We addressed the following research question: From the perspective of researchers and YPCC, what is considered meaningful impact of PPI in research?

## 2 MATERIALS AND METHODS

### 2.1 Design

Q methodology combines qualitative and quantitative techniques to systematically study how people think about a certain topic.^23^, ^24^, ^25^ In Q methodology, between 40 and 60 participants are presented with a sample of 20–100 statements, which they rank order onto a grid. After this, they are asked to reflect on the choices they made. Factor analysis is then conducted to reveal patterns of similarity in how statements were sorted by respondents. Unlike conventional factor analysis, in Q methodology, the individual rankings—not the different statements—are taken as variables. The resulting factors represent groups of individuals with similar perspectives. Participants’ reflections on their rankings are used to interpret and describe the factors.

We chose Q methodology because it was very suitable to answer our research question for several reasons. First, it can be used to systematically study perspectives and compare their similarities and differences.^24^, ^25^ Second, it also prompts participants to carefully weigh the importance of various statements, as the method compels them to make a choice.^24^, ^26^ Third, it combines the strengths of quantitative and qualitative research. The factors retrieved in quantitative analyses are given meaning by using participants’ reflections on their sorting of statements.^24^, ^25^

### 2.2 Participants

The participants in the study were researchers and YPCC between 15 and 30 years old. Participants were required to have at least some experience with PPI of YPCC. Researchers were recruited through the authors’ networks in the academic field. YPCC were recruited through the network of an adolescent with a chronic condition who was involved in the preparation of this study and an announcement placed on the website of JongPIT, a Dutch foundation for and led by YPCC. Snowballing was used to contact additional researchers and YPCC.

After recruitment, 50 participants sorted statements and were interviewed. However, four participants were excluded from the analysis. During data collection, it became clear that one researcher and one YPCC had not fully understood the sorting task. Two YPCC were not familiar with PPI. Consequently, the final sample consisted of 46 participants: 20 YPCC and 26 researchers. All researchers and YPCC were Dutch and therefore mostly had experience with PPI in the Netherlands. There were two exceptions: one Dutch researcher conducted research in Denmark and another in Canada. Researchers were not reimbursed for their participation in the study; YPCC received a gift certificate.

### 2.3 Data collection

Statements were collected about various types of impact, such us ‘Young people acquire new knowledge and skills’ and ‘PPI contributes to a society in which everyone can participate’. A recently conducted literature review was used as the primary input to formulate statements^4^ by scanning the data on reported motivations and benefits of PPI. This was performed by the first author. During this process, she merged some statements that were very similar. This resulted in a list of 39 statements, which was discussed by all authors and the adolescent with a chronic condition who was involved in the preparation of this study. In an iterative process, it was decided to remove some additional statements to eliminate repetition. In addition, statements were clarified and shortened to improve comprehensibility. The final selection (also called the Q sample) consisted of 33 statements (Table 2).

**Figure 1:**
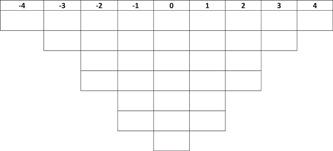
Q sort table

Participants were asked to rank order the Q sample using a *Q sort table* (Figure 1). Due to the COVID‐19 circumstances, they were invited to do this digitally. Participants were sent a link to the ranking exercise, which was programmed using the VQMethod.^27^ The ranking exercise started with an overview of all 33 statements. First, they were asked to sort the statements (options: agree, neutral, disagree) guided by the question ‘What are your motivations for doing PPI?’ The second step was to rank all statements in the Q sort table. Based on the first sorting exercise, participants were instructed to rank the statements based on how much they agreed with them. The more they agreed with a statement, the more they placed it to the right of the Q sort table. The more they disagreed with a statement, the more they placed it to the left. The statements they agreed or disagreed with most were placed at the extreme right (+4) and left (−4). After finishing the Q sort table, participants were asked to answer a few short questions about their background and previous experience with PPI of YPCC (‘little’, ‘some’ or ‘much’).

After the ranking exercise, telephone interviews or video calls were conducted with participants to reflect on their individual ranking of statements. Participants answered questions about the two or three statements that they placed at the extreme right (+3 and +4) and left (−3 and −4) sides. Some additional questions about successful PPI and impact were included to ensure that all relevant topics regarding meaningful impact were addressed (Box 1). All interviews lasted a maximum of 45 min. They were audio‐taped and transcribed.

BOX 1. **Interview guide**

1. Can you tell me something about your experiences with PPI?
2. Before this interview you did a sorting task. How did this go? How did you make choices?
3. You sorted … as the statement you agreed with very much/the most. Why?
4. You sorted … as the statement you agreed with very little/the least. Why?
5. What does successful PPI mean to you? What is meaningful impact?

### 2.4 Analysis and interpretation

The individual rankings of statements—also termed Q sorts—were analysed using principal component analysis, followed by Varimax rotation. Analyses were conducted in Stata 15.0 using qfactor.^28^ The selection of a factor solution was based on both quantitative and qualitative criteria.^23^, ^29^ Some statistical features were examined. For example, only factors with an eigenvalue in excess of 1.00 were selected. Another requirement was that at least two Q sorts must load onto each factor. Ideally, the percentage of explained variance in the chosen factor solution is 35%–40% or higher.^29^ In our analyses, factor solutions with 2–7 factors fulfilled these requirements.

Since factors should be interpretable and represent coherent and comprehensible narratives, the final factor solution was based on the qualitative interviews.^29^ As a part of Q methodology, the views expressed during the interviews were compared with the idealized Q sort of each factor, which are called the composite sorts. In a composite sort, ‘the Q sorts of all participants who define a given factor are merged together to yield a single (factor exemplifying) Q sort’.^23^ The choice for the final factor solution was based on the extent to which composite sorts were consistent with the qualitative reflections of the participants whose Q sorts defined each factor.

Interviews were analysed in MaxQDA. The statements of the ranking exercise were translated into codes. All fragments reflecting on specific statements were coded within the corresponding code. This resulted in an overview of how participants interpreted different statements and how they reflected on them. The principal author compared the interpretations and reflections of participants and assessed the extent to which they represented similar perspectives on impact. Based on this, a preliminary factor solution was chosen, which was discussed with the other authors. The discussion focused on whether the factor solution covered all perspectives on impact and how they related to each other. Ultimately, consensus was reached on the definitive factor solution.

Interpretation of the factor solution was based on the expressed views and elaborations in the interviews and the relative placement of all statements within each composite sort. Particular attention was paid to characterizing and distinguishing statements. Characterizing statements are statements on or near the extremities of the Q sort table (+4, +3, −3 and −4). Distinguishing statements are statements that are placed on significantly different positions in the Q sort tables of different composite sorts.

### 2.5 Validity

All participants provided informed consent before they started the ranking exercise. The study was conducted in accordance with the General Data Protection Regulation. Formal ethical approval of this study was not required under the prevailing Dutch legislation.

## 3 RESULTS

### 3.1 Description of participants

Table 1 presents a summary of the characteristics of the study participants. On average, YPCC were 23 years old (range 17–29 years). Their experience with PPI varied from little or some (50%) to much (50%). The researchers who participated were on average 39 years (range 22–63) of age. Their experience in doing research varied, with interns, PhD students, postdocs, senior researchers and professors taking part in the study. They had little or some (62%) or much (38%) experience with PPI of YPCC.

**Table 1:** Study participants

	Young people with a chronic condition		Researchers	
	*N*	% or *M* (min–max)	*N*	% or *M* (min–max)
Total	20	100	26	100
Sex				
Female	12	60	23	88
Male	8	40	3	12
Age (years)	20	23 (17–29)	26	39 (22–63)
Education^a^				
Elementary school	0	0	0	0
Secondary education	3	15	0	0
Postsecondary education	17	85	26	100
Experienced in PPI of young people with a chronic condition (%)				
Little or some experience	10	50	16	62
Much experience	10	50	10	38

Abbreviation: PPI, Patient and Public Involvement.

^a^ Highest education currently enrolled in or completed.

### 3.2 Perspectives on meaningful impact

Four factors and composite sorts were extracted from the data, that is four distinct perspectives on meaningful impact of involving YPCC in research. These factors were defined by 37 Q sorts (80%); 9 Q sorts did not load significantly onto any of the factors. Table 2 shows the position of each statement in the composite sorts.

**Table 2:** Statements (Q sample) and their position in the four composite sorts

		Factor A	Factor B	Factor C	Factor D
1	Research involving young people receives more media attention	−3^a^	−3^a^	−2^a^	−4^a^
2	Young people help researchers to better understand research outcomes	1	4^a^	−1	0
3	Young people describe research outcome in a language young people understand	1	1	0^a^	1
4	When a peer describes research, young people can make a more informed decision about research participation	0	0	−2	−1
5	It improves the usefulness of research outcomes in practice	3	2	3	1^a^
6	Researchers become more creative	1	1	2	0^a^
7	It is useful for young people's resume	−2^a^	−2^a^	1	0
8	It motivates young people to participate in democracy	−1^a^	−4^a^	−3^a^	2^a^
9	It is increasingly a requirement of funders and scientific journals that young people are involved in research	−4	−1	−1	−3
10	Young people feel heard	0	3	0	3
11	It increases equality between young people and researchers	0	−2^a^	−3^a^	0
12	Young people are becoming more interested in research	−1	−1	−1	0
13	Young people gain new insights on themselves	−1	−1	1^a^	0
14	Researchers obtain new insights from young people	2	3	2	1^a^
15	Young people get to know other young people	−3	0^a^	0^a^	−3
16	Young people enjoy their involvement	−2^a^	1^a^	0^a^	3^a^
17	Researchers enjoy young people's involvement	−1	1^a^	−2	−1
18	It contributes to a society in which everyone can participate	0^a^	−3^a^	4^a^	2^a^
19	Young people build a network	−2	−2	1^a^	−1^a^
20	Young people are given the opportunity to do something useful for other young people	0^a^	1^a^	2	2
21	Young people know which research methods suit the target group	1^a^	−1	−4^a^	−2
22	Young people come up with research questions that are important to young people	4^a^	2	0^a^	1
23	Young people put young people who participate in the study at ease	0	0	−1	−1
24	Researchers learn who exactly their research is about	2	2	2	1
25	Young people have the right to participate in research that concerns them	2^a^	0^a^	3^a^	4^a^
26	Young people and researchers build valuable relationships	−1	0	0	1
27	It helps young people to think about their future	−2	−2	−1^a^	−2
28	Young people acquire new knowledge and skills	0^a^	2	1	2
29	Researchers look beyond just their own experiences	3^a^	0	1	−1^a^
30	Young people spread the results of research more easily among young people	1^a^	0	−2	−2
31	Young people ask questions in interviews and questionnaires that researchers do not come up with	2^a^	1	0	0
32	It is easier for young people to recruit young people as participants for research	1^a^	−1	−1	−2
33	Young people gain self‐confidence	−1	−1	1^a^	−1

^a^ Statement is placed on a significantly different position compared to the other composite sorts.

Below, the factors are described in more detail. Each factor description starts with general information about statistics and what participants loaded onto this factor. Next, the factor interpretation is provided. In the text, we used parentheses to provide details about statements and their position in the composite sorts. For example (5: +3) means that statement 5 was positioned at +3 in the composite sort.

### 3.3 Factor A: PPI improves the quality of research

#### 3.3.1 General information

The Q sorts of 12 participants significantly loaded onto Factor A. This explains 19% of the study variance and has an eigenvalue of 14.8. Eleven of the participants were researchers and one was a YPCC.

#### 3.3.2 Factor interpretation

The quality of research is central to Factor A. PPI improves the quality of research by helping researchers to look beyond their own experiences (29: +3). As one researcher explained, ‘researchers focus on the details of their research, but can sometimes lose the bigger picture, what they are doing it for’. The benefits for research can take many forms. Most importantly, YPCC come up with research questions that matter to YPCC (22: +4). This can improve the relevance of research and prevent a ‘mismatch’ between what is studied and what should be studied according to YPCC to improve their quality of life. Furthermore, YPCC ask questions in interviews and questionnaires that researchers do not come up with themselves (31: +3). They can also help to recruit other YPCC as research participants (32: +1) and they know which research methods are suitable for YPCC (21: +1). At the end of a research project, they help in disseminating research outcomes (30: +1). Given these benefits, PPI can eventually increase the usefulness of research in practice (5: +3). As one researcher explained: ‘Knowledge becomes more useful, because it is more in line with their experiences and the questions they have. It can be integrated more easily into practice, because it arises from practice’. Since their PPI can contribute to better research addressing their issues and questions, YPCC are entitled to be involved in research concerning them (25: +2). One researcher who conducts research in a hospital stated: ‘we have a moral obligation to involve young people, because all of us can actually contribute to the improvement of health care’. Although PPI can also have individual benefits for the YPCC who are involved in research, this is not an aim of PPI. For example, it can improve YPCC's knowledge and skills (28: 0), contribute to their resume (7: −2) or provide them with new insights about themselves (13: −1). These are, however, mainly ‘side‐effects’.

### 3.4 Factor B: PPI facilitates dialogue and understanding

#### 3.4.1 General information

Factor B has 11 significantly loading Q sorts of participants and explains 15% of the study variance. It has an eigenvalue of 5.0. Eight participants were researchers, and three participants were YPCC.

#### 3.4.2 Factor interpretation

Factor B focuses on engaging in a dialogue and improving researchers’ understanding of YPCC's reality. Discourses between YPCC and researchers can help researchers to better understand research outcomes (2: +4). As one young person with a chronic condition stated: ‘It is hard for researchers to understand how we think. They are not the same age and they have not been through the things we have been through. We can help them understand’. It is therefore imperative to make sure that YPCC are and feel heard (10: +3). ‘It helps to see through their eyes and to understand what they wish for, find and think, and what is going on in their lives’. Engaging in a dialogue with YPCC can provide researchers with a lot of new insights (14: +3). This can help to ‘place research outcomes in context’ and eventually ‘improve research quality’. However, it seldom leads to changes on the societal level. The aim of PPI is not to motivate YPCC to participate in democracy (8: −4) or to contribute to an inclusive society (18: −3). As one researcher said: ‘Although they may grow as a citizen or a human being, that is still a long way off’. Benefits for YPCC, such as building a resume (7: −2) and a network (19: −2), are seen as ‘side‐effects’. Equality between YPCC and researchers is not necessarily an aim (11: −2). ‘It is about different roles. YPCC bring unique knowledge and experiences to the table. I wouldn't talk about equality or inequality, just different perspectives’.

### 3.5 Factor C: PPI contributes to an equal and inclusive society

#### 3.5.1 General information

Six Q sorts of participants significantly loaded onto Factor C. This explains 11% of the study variance and has an eigenvalue of 3.1. Five participants were YPCC, and one participant was a researcher.

#### 3.5.2 Factor interpretation

Central to Factor C is equality and inclusivity. PPI contributes to a society in which everyone can participate (18: +4) and is therefore a right of all YPCC (25: +3). YPCC can contribute to the usefulness of research in practice (5: +3). ‘And when research is useful in practice, you can really achieve something’. PPI enables YPCC to do something valuable for other YPCC who are in a similar situation (20: +2). PPI also contributes to an inclusive society, since it propagates the message of ‘equality’ and ‘inclusivity’. As one young person stated: ‘It is important that everyone can participate, despite their limitations. You should focus on the things you can do. When you involve young people in research, they can show that a lot is possible’. In line with this, PPI can contribute to the personal growth of YPCC. They can gain new insights about themselves (13: +1), become more self‐confident (33: +1) and build a network (19: +1). One young person stated: ‘Rewards for PPI can also be that you can put something on your resume or that you learn new things. I think this may be even more valuable than receiving money or whatever’. Although PPI should contribute to a more inclusive and equal society, YPCC and researchers are not equal in research processes (11: −3). Researchers are experienced in doing research and bear more responsibility. Therefore, they have the final say in decisions related to the research. ‘Researchers are still far above young people. In my experience, young people are mostly there to provide input’.

### 3.6 Factor D: YPCC have a right to make their voices heard

#### 3.6.1 General information

Eight Q sorts of participants loaded significantly onto factor D. This explains 11% of the study variance. It has an eigenvalue of 2.6. Seven participants were YPCC, and one participant was a researcher.

#### 3.6.2 Factor interpretation

Factor D focuses on YPCC's right to make their opinions heard. YPCC have the right to be involved in research that concerns them (25: +4). As one young person explained: ‘Young people have relatively little to say. They don't really feel taken seriously in society and politics. And if they are involved in research and taken seriously, then that is at least a start’. Hearing YPCC's voices in research (10: +3) is a first step towards them playing a more active role in the democracy (8: +2). When YPCC are provided with real opportunities to express themselves, research involvement is a fun experience, as stated by this researcher: ‘It should be fun, make them enthusiastic and give them energy. They are participating because they have something to say to the world’. PPI provides opportunities to do something useful for others (20: +2) and to contribute to an inclusive society (18: +2). On a smaller scale, PPI can contribute to the usefulness of research results (5: +1), provide researchers with new insights (14: +1) or improve research quality, for example, by coming up with important research questions (22: +1). However, these are definitely not the aims of PPI; it is above all a right, and the focus should be on providing YPCC with sufficient space to make their opinions heard rather than improving the research as such.

## 4 DISCUSSION

In the present study, we examined the perspectives of researchers and YPCC on meaningful impact of PPI in research. The resulting insights offer valuable opportunities for improving the evaluation of PPI of YPCC. This enables advancement in a field that has shown limited progress in recent decades.

Using Q methodology, four distinct perspectives on meaningful impact were identified: (A) improving research quality, (B) facilitating dialogue and understanding, (C) contributing to equality and inclusivity and (D) doing justice to YPCC's rights. The perspectives show differences as well as similarities. Distinctive in perspective A is the focus on improving how research is designed and conducted. Conducting research that will improve the lives of YPCC is considered the most important type of impact, from this perspective. In perspective B, the highest value is placed on improved understanding of research outcomes and formulating correct conclusions and implications. One similarity between perspectives A and B is the emphasis on improving the usefulness of research. In perspective C, the most important type of impact is a more inclusive society. In contrast to perspectives A and B, the personal development of the YPCC involved in research is also an aim. Perspective D prioritizes the right of YPCC to be involved above all other impacts.

The findings demonstrate that researchers and YPCC generally have different perspectives on meaningful impact of PPI. We are indeed aware that Q methodology is not particularly suited to make claims about the division of perspectives among different groups. As Watts and Stenner explained, Q methodology is less about ‘*who* said what about X’ than ‘*what* is currently being said about X’.^23^ Nevertheless, it is notable that perspectives A (improving research quality) and B (facilitating dialogue and understanding) are predominantly based on the Q sorts of researchers, while perspectives C (contributing to equality and inclusivity) and D (doing justice to YPCC's rights) are predominantly based on the Q sorts of YPCC. This finding highlights the importance of taking into account all perspectives in evaluations of PPI.

The findings also show that a difference can be made between meaningful impact that researchers and YPCC strive for and (unintended) secondary impact. It is important to make this distinction in PPI evaluations to improve critical reflection. This can be done by predetermining indicators of meaningful impact. However, we noticed during data collection that many participants were not used to defining which impact matters most to them. A similar experience was reported by Cook et al.^21^ The perspectives emerging from this study can therefore serve as an aid in specifying meaningful impact in PPI evaluations. We should also note that that the perspectives are not meant for categorizing researchers and YPCC into different groups. They are the result of various merged Q sorts and qualitative interpretation, so not all researchers and YPCC may identify completely with them. Rather, the perspectives should be used to create awareness that meaningful impact can mean different things for different individuals.

The perspectives and statements can also serve as an aid for operationalizing meaningful impact into measurable indicators. Many researchers consider it very difficult to quantify PPI impact.^30^ However, many types of impact can be operationalized based on the statements used in this study. For example, indicators could focus on the extent to which YPCC consider the research question important (Statement 22), the extent to which researchers obtained new insights (Statement 14), the new knowledge and skills acquired by being involved (Statement 28) and the extent to which YPCC felt they were able to exercise their right to have a say in research about them (Statement 25). Some types of impact are more difficult to quantify, such as a more inclusive society (Statement 18). For these types of impact, more flexible and creative operationalizations are needed that describe them in the best way possible. For example, measuring a more inclusive society may be approached by asking the YPCC involved or those using the study results in practice about their perspectives on the achievement of this impact.

One of the retrieved perspectives focuses on PPI as a right of YPCC. It has sometimes been suggested that impact evaluations are not useful in this context, since the focus is on YPCC exercising their rights rather than on impact.^5^, ^31^ However, in line with Staley, we believe that impact evaluations can always provide relevant feedback on the success of a PPI approach and the achievements of its aims.^5^ Staley emphasized that understanding PPI as a right can give patients ‘a seat at the table’, whereas impact evaluations can improve PPI approaches to ensure that they can actually exercise their rights. Although less explicit, those who are doing PPI based on moral reasons also strive for impact. They wish for PPI to increase YPCC's opportunities to have a say in matters that affect them.

We also noticed during the interviews that questions about impact were answered with caution and many qualifications. Researchers in particular feared that impact would become a condition for doing PPI. We would suggest, however, to look at impact evaluations as a learning mechanism and a way to obtain insight into the best ways to do PPI.^5^ According to Lundy's line of reasoning, imperfect PPI is not the end but rather the beginning of a learning process.^32^ When PPI does not result in the desired impact, much can be learnt from critical reflection on questions such as ‘why not?’ and ‘what can we do differently next time?’

### 4.1 PPI in the current study

An adolescent with a chronic condition collaborated in the preparation of the current study. Our collaboration was shaped based on what Franks called ‘Pockets of PPI’.^33^ We divided the study into different parts and discussed in what parts the adolescent wished to be involved. Due to COVID‐19 circumstances and the nature of her chronic condition (a hearing impairment), our collaboration took place via email. The adolescent was reimbursed for her involvement.

The adolescent provided input on the statements for the sorting task. She suggested that we add ‘YPCC feel heard’. This appeared to be a relevant addition, since many study participants rated this statement as important. Based on the suggestions of the experience expert, we also reformulated statements, such as ‘YPCC are given the opportunity to do something useful for *others*’, which was changed to ‘YPCC are given the opportunity to do something useful for *other YPCC*’. The adolescent also helped in recruiting YPCC as study participants via her network.

To make sure that the perspectives of YPCC were also included in the final stages of the study, we informally discussed our findings with several other adolescents with a chronic condition who were involved in another research project.

### 4.2 Strengths and limitations

To the best of our knowledge, this is the first study that has examined the impact of PPI in so much detail. Q methodology enabled us to systematically study perspectives and compare their similarities and differences. An important strength of this method was that it compelled participants to choose which impact matters to them most. This ensured that they carefully considered their motivations in a way they had not done before.

Due to COVID‐19 circumstances, we had to conduct the study online. Although the programme VQMethod^27^ was very suitable for doing the sorting task online, one limitation was that participants could not complete the sorting tasks in the presence of the researchers. This resulted in the exclusion of a few participants who did not fully understand the sorting task. Also, it would have been valuable to hear their thoughts and considerations during the sorting tasks rather than retrospectively during the interviews. Another limitation is that recruitment of participants through the networks of the authors may have resulted in some selection bias. However, by applying snowballing, it was ensured that researchers and YPCC outside the networks were also reached. Finally, it could have been valuable to discuss the sorting tasks in focus groups rather than interviews to enrich our understanding of overlap and differences between arguments for doing PPI. The interviews, however, enabled us to hold more in‐depth discussions with all participants about what motivated them.

The current study provides extensive insight into perspectives on meaningful impact of researchers and YPCC. It would be interesting for future studies to explore these perspectives in different age groups, such as children, adults and the elderly, since it is conceivable that desired impact may differ according to age.

## 5 CONCLUSIONS

In recent decades, limited progress has been made in evaluating the impact of PPI with YPCC. In the study presented here, we aimed to change this by clarifying the concept of meaningful impact. Using Q methodology, we identified four distinct perspectives on meaningful impact among researchers and YPCC: improving research quality, facilitating dialogue and understanding, achieving equality and inclusivity and doing justice to YPCC's rights. Researchers and YPCC generally have different perspectives on meaningful impact. Evaluations should therefore take into account the perspectives of everyone involved in a PPI process. Our study also highlights the importance of predetermining indicators for meaningful impact in PPI evaluations. The perspectives retrieved in this study can serve as a starting point for this and for operationalizing them into measurable indicators.

## CONFLICT OF INTERESTS

The authors declare that there are no conflict of interests.

## ETHICS STATEMENT

All participants provided informed consent. The study was conducted in accordance with the General Data Protection Regulation. Further ethical approval of this study was not required under the applicable Dutch legislation.

## Data Availability

The data that support the findings of this study are available from the corresponding author upon reasonable request.
